# Compound Enzyme Supplementation Improves Intestinal Health, Immunity, and Antioxidant Capacity in Pigeons via Gut Microbiota and Metabolome Modulation

**DOI:** 10.3390/ani16152353

**Published:** 2026-08-02

**Authors:** Jiahao Yan, Ying Peng, Tiantian Gu, Li Chen, Wenwu Xu, Yong Tian, Jindong Ren, Rongyang Li, Jiayi Su, Lihong Gu, Jihui Wen, Lizhi Lu, Tao Zhang, Tao Zeng

**Affiliations:** 1 College of Animal Science and Technology, Yangzhou University, Yangzhou 225009, China; mz120241632@stu.yzu.edu.cn; 2 Institute of Animal Husbandry and Veterinary Science, Zhejiang Academy of Agricultural Sciences, Hangzhou 310021, China; lypengying@163.com (Y.P.); gutt@zaas.ac.cn (T.G.); chenli@zaas.ac.cn (L.C.); xuww@zaas.ac.cn (W.X.); tyong@zaas.ac.cn (Y.T.); renjd@zaas.ac.cn (J.R.); lirongyang@zaas.ac.cn (R.L.); sjy@zaas.ac.cn (J.S.); lulz@zaas.ac.cn (L.L.); 3 Institute of Animal Science and Veterinary Medicine, Hainan Academy of Agricultural Sciences, Haikou 571100, China; gulihong@hnaas.org.cn; 4 Aofeng Pigeon Industry Co., Ltd., Pingyang County, Wenzhou 325403, China; wenjihui888@163.com

**Keywords:** compound enzyme preparations, antioxidant capacity, immune response, intestinal health, pigeon

## Abstract

The study evaluated the effects of dietary compound enzyme preparations (CEPs) supplementation on antioxidant capacity, immune response, and intestinal health of pigeons. A total of 288 White King parent pigeons were randomly assigned to four treatments, with six replications of 12 pigeons each. The control (CK) group received a basal diet, whereas the CEP groups were fed a basal diet with 0.25 g/kg (low compound enzyme preparation group, LCEP), 0.5 g/kg (middle compound enzyme preparation group, MCEP), and 1.0 g/kg (high compound enzyme preparation group, HCEP) CEPs. Results showed that supplementation with 0.25 g/kg of CEPs significantly increased duodenal crypt depth (CD), as well as jejunal villus height (VH) and the villus height-to-crypt depth ratio (VCR) (*p* < 0.05). It also increased the immunoglobulins of serum, such as IgA, IgG, and IgM, as well as enhanced antioxidant capacity by increasing superoxide dismutase (SOD), catalase (CAT), and glutathione peroxidase (GSH-Px) activities of serum (*p* < 0.05). Furthermore, we found that CEPs altered ileum content microbiota composition, increasing the relative abundance of *p_Bacteroidota* and *g_Bifidobacterium* (*p* < 0.05). In addition, untargeted metabolomics analysis identified 92 upregulated and 95 downregulated metabolites. Pathway enrichment analysis of these differential metabolites revealed that CEP supplementation altered the metabolomic profile of the ileum contents, with differential metabolites mainly enriched in alanine, aspartate and glutamate metabolism, fatty acid biosynthesis, and unsaturated fatty acid biosynthesis pathways. These results demonstrated that dietary supplementation with CEPs at different doses improved antioxidant capacity to varying degrees and maintained intestinal immune balance by modulating microbiota, which provides a theoretical basis for the rational application of compound enzyme preparations in breeding pigeon feed. Moreover, this study provides mechanistic evidence that CEPs can improve intestinal health, immunity, and antioxidant capacity. It also fills a key knowledge gap in pigeon nutrition by linking enzyme use with gut microbiota and metabolic regulation.

## Simple Summary

In this study, we evaluated the effect of the compound enzyme preparations (CEPs) on intestinal morphology, antioxidant capacity, immune function, intestinal microbiota, and intestinal metabolites. By combining serum biochemical analysis, histomorphological assessment, 16S rRNA gene sequencing, and untargeted metabolomics analysis, we find that CEPs, especially at 0.25 g/kg, significantly enhanced the integrity of the intestinal barrier, the secretion of immunoglobulins, and the diversity of microorganisms. Furthermore, the regulation of key metabolic pathways caused by CEPs, including amino acid metabolism, fatty acid synthesis, and one-carbon metabolism, provides a mechanistic explanation for how enzyme supplementation promotes intestinal health.

## 1. Introduction

Due to the shortage and rising cost of corn supply, wheat has become an important feed ingredient in poultry diets [1]. Currently, pigeon diets are mainly composed of plant-based raw materials such as grains. These raw materials contain a large amount of non-starch polysaccharides (NSPs), such as cellulose, hemicellulose, β-Glucans and arabinoxylans, which form the plant cell walls [2]. The dense network structure formed by these NSPs can encapsulate starch, protein, and other nutrients, limiting the access of digestive enzymes with nutrients. Therefore, due to the lack of enzymes capable of degrading NSPs, nutrient digestibility is reduced [3]. In addition to affecting the utilization of nutrients, soluble NSPs will also increase the viscosity of intestinal food, thus limiting the diffusion of nutrients and reducing the interaction between digestive enzymes and their substrates [4]. Supplementing exogenous enzymes can hydrolyze NSPs into smaller oligosaccharides, thus reducing the viscosity of food, improving the digestibility of nutrients, reducing intestinal inflammation and oxidative stress, enhancing intestinal immune function, and supporting the maintenance of intestinal normal form and absorption function [5].

The small intestine is composed of three consecutive areas, which are the duodenum, jejunum, and ileum in the pigeons’ digestive system [6]. Each section is involved in the efficient digestion and absorption of feed nutrients. The duodenum receives the secretions from the liver and pancreas, which facilitate the digestion of fats, proteins, and carbohydrates into absorbable monomers [7]. The jejunum is the primary site of nutrient absorption, where a well-developed villus–crypt structure and abundant membrane transporters facilitate the uptake of glucose, amino acids, fatty acids, vitamins, and minerals [8]. The ileum completes nutrient absorption and is particularly important for the reabsorption of bile acids, thereby sustaining enterohepatic circulation and lipid digestion efficiency, while also contributing to intestinal immune defense through gut-associated lymphoid tissue [9].

Exogenous enzyme preparations added to the diet can hydrolyze NSPs, thereby promoting the digestion and utilization of nutrients. These enzyme preparations can be classified into two types based on their composition: single enzyme preparations (SEPs) and compound enzyme preparations (CEPs). The SEPs usually contain only one type of enzyme and act on specific substrates such as cellulose; thus, their effect on digestion and absorption is limited. The CEPs are products formulated from multiple sources of enzymes and can simultaneously degrade various substrates in the feed, thereby enhancing the nutrient utilization; their effects are generally superior to those of SEPs [1]. Previous studies have shown that adding 200 mg/kg of CEPs to the diet significantly increases serum IgG, TP, and ALB levels in broilers [10]. Based on these findings, we speculate that CEPs may exert similar effects in pigeons.

We paid particular attention to ensuring that the enzyme composition of the CEPs matched the NSPs in diets. Enzymes such as xylanase and cellulase target arabinoxylans and cellulose, while proteases and amylases enhance protein and starch digestion, respectively. This targeted enzymatic strategy is expected to reduce NSP-induced nutrient encapsulation, improve intestinal function, and promote a more favorable microbial ecosystem.

The existing literature provides limited information on nutrient regulation strategies in pigeon diets, particularly regarding the relationship between the effects of CEPs and the intestinal function of pigeons. Therefore, we hypothesized that dietary supplementation with CEPs may improve intestinal health in pigeons by modulating the gut microbiota and associated metabolic profiles. Based on this hypothesis, this study aimed to evaluate the impact of varying CEP concentrations on intestinal histopathology, gut microbiota, and gut metabolites associated with antioxidant capacity and immune response.

## 2. Materials and Methods

### 2.1. Animals

A total of 288 2-year-old female white feathered pigeons were selected as experimental animals with a weight range of 500–600 g. All pigeons were managed under identical husbandry conditions and entered and exited the production system simultaneously; therefore, their health and physiological state were considered comparable. They were randomly allocated into four groups using a random number table and a computer-generated randomization sequence, with six replicates per group and 12 pigeons per replicate. The experiment was conducted entirely at the commercial farm of Huzhou Huajia Special Breeding Co., Ltd. All pigeons were housed in environmentally controlled three-tier cages (60 cm × 50 cm× 45 cm; two birds per cage) under an ambient temperature of 25 °C and a relative humidity of 65%, and were provided with 10 h of artificial light per day. We implemented a whole-grain feeding strategy, and the CEP was mixed with the health sand. The basal diet and the health sand were provided in separate feeders, allowing the pigeons free and simultaneous access to both. The control (CK) group was fed with a basal diet, while the low compound enzyme preparation, middle compound enzyme preparation, and high compound enzyme preparation groups were respectively supplemented with 0.25, 0.5, and 1.0 g/kg of a compound enzyme preparation in the health sand. The pre-test period for the pigeons was 7 days, and the test period was 28 days.

### 2.2. Basal Diet

The basal diet was formulated to meet the nutrient requirements of pigeons and consisted primarily of corn (53.0%), pea (30.0%), wheat (7.0%), and sorghum (7.0%). The calculated metabolizable energy (ME) and analyzed crude protein (CP) contents were 12.81 MJ/kg and 12.90%, respectively, while the diet contained 1.20% calcium, 0.54% available phosphorus, 1.05% lysine, and 0.44% methionine. The complete ingredient composition and nutrient levels are presented in Table 1. The cereal- and legume-based ingredients in the basal diet provide naturally occurring non-starch polysaccharides (NSPs), which serve as substrates for the supplemented compound enzyme preparation.

### 2.3. CEP

The enzyme activity profile of CEP is as follows: cellulase ≥ 200 U/g, xylanase ≥ 9000 U/g, amylase ≥ 100 U/g, protease ≥ 3000 U/g, β-glucanase ≥ 1000 U/g, β-mannanase ≥ 400 U/g, and pectinase ≥ 200 U/g. and is produced by Cangzhou Xiaosheng Enzyme Biotechnology Co., Ltd. (Cangzhou, Hebei, China); the instruction manual of the CEP is provided in the Supplementary Materials.

### 2.4. Sample Collection

At the end of the experiment (35 days), one pigeon from each replicate per group were randomly selected, and approximately 1.5 mL of blood was collected from the wing vein to obtain serum. The pigeons were deeply anesthetized prior to euthanasia, and euthanasia was performed by decapitation to minimize pain and distress, and 1.5 cm of the duodenum, jejunum, and ileum were taken and fixed in 4% paraformaldehyde for histomorphology. Then, the ileal contents were collected immediately and kept at −80 °C for subsequent tests.

### 2.5. Morphological Analysis of Intestinal Tissues

Samples collected were processed into 5 μm thick sections. For histological examination, sections were stained with hematoxylin and eosin (H&E), then visualized and imaged using an Olympus microscope (Olympus, Tokyo, Japan). After HE staining and imaging, the VH and CD were measured in K-Viewer 1.7.1.1 (Yuyao, Hangzhou, China), and the VH/CD ratio was calculated.

### 2.6. Serum Biochemical Index Determination

The concentrations of total protein (TP), albumin (ALB), globulin (GLB), aspartate aminotransferase (AST), and alanine aminotransferase (ALT), as well as the immune indicators immunoglobulin A (IgA), immunoglobulin G (IgG), and immunoglobulin M (IgM) in serum samples were detected following the instructions (Beijing Huaying Biotechnology Institute, Beijing, China). Commercial assay kits for the superoxide dismutase (SOD), catalase (CAT), total antioxidant capacity (T-AOC), glutathione peroxidase (GSH-Px), and malondialdehyde (MDA) were all supplied by Beijing Huaying Biotechnology Research Institute to determine the antioxidant capacity.

### 2.7. Untargeted Metabolomics Analysis

Each treatment group consisted of six replicates with 12 pigeons per replicate. One ileal content sample was randomly selected from each replicate for 16S rRNA sequencing and metabolomics analyses.

This study employed a non-targeted metabolomics approach based on liquid chromatography-mass spectrometry (LC-MS) technology for sample analysis. The extraction process of metabolites was as follows: 20 mg of sample was accurately weighed and homogenized with pre-cooled methanol solution containing 5 ppm of L-2-chlorophenylalanine (internal standard). The sample was then ground with a high-throughput tissue grinder, ultrasonically extracted, and left to stand at low temperature. After centrifugation at 4 °C and 12,000 rpm, the supernatant was filtered through a 0.22 μm micropore filter and used for instrument detection. Chromatographic separation was performed on a Thermo Vanquish Flex ultra-performance liquid chromatography system (Thermo Fisher Scientific, Waltham, MA, USA) using an ACQUITY UPLC HSS T3 column, with the column temperature maintained at 40 °C. The mobile phase consisted of 0.1% formic acid in water and 0.1% formic acid in acetonitrile, and effective separation of metabolites was achieved within 8.5 min through a gradient elution program at a flow rate of 0.4 mL/min and an injection volume of 2 μL. Mass spectrometry detection was carried out on a Thermo Orbitrap Exploris 120 high-resolution mass spectrometer (Thermo Fisher Scientific, Waltham, MA, USA) using an electrospray ionization source, with data collected in both positive and negative ion modes. Raw data were processed using MS-DIAL software (version 4.9.221218) for peak extraction, alignment, missing value imputation, and normalization. Metabolite identification was based on the PSNGM integrated database of Pasonno, which includes multiple public and self-built spectral libraries such as mzCloud, HMDB, and LIPID MAPS.

Quality control (QC) samples were prepared by pooling equal aliquots of extracts from all study samples and were injected periodically throughout the analytical sequence to monitor the stability and reproducibility of the LC-MS system. Data quality was assessed based on total ion chromatogram (TIC) overlap, Pearson correlation analysis among QC samples, relative standard deviation (RSD) evaluation, and principal component analysis (PCA). Features with an RSD greater than 30% in QC samples were excluded to ensure data reliability. The metabolomics data were normalized using the total peak area normalization method. According to the Metabolomics Standards Initiative (MSI) guidelines, all reported metabolites were assigned an identification confidence level of Level 2 or higher.

### 2.8. Analysis of 16S rRNA Sequencing of Intestinal Microbiota

Total genomic DNA was extracted from samples using the MagBeads FastDNA Kit (116564384) (MP Biomedicals, Santa Ana, CA, USA). DNA quality and concentration were assessed by agarose gel electrophoresis and NanoDrop spectrophotometry (Thermo Fisher Scientific, Waltham, MA, USA). Bacterial 16S rRNA gene regions of microbial marker genes were amplified using high-fidelity Q5 DNA polymerase with 16S V3V4 region (F: ACTCCTACGGGAGGCAGCA; R: GGACTACHVGGGTWTCTAAT). PCR products were verified by agarose gel electrophoresis, purified, quantified using the Quant-iT PicoGreen dsDNA Assay Kit (Thermo Fisher Scientific, Waltham, MA, USA). Amplicon libraries were constructed using the Illumina TruSeq Nano DNA LT Library Prep Kit (Illumina, San Diego, CA, USA), followed by quality control and quantification. Paired-end sequencing was then performed on an Illumina platform. Raw sequencing data were processed using QIIME2 (v2024.5) for demultiplexing, primer trimming with Cutadapt, quality filtering, denoising, chimera removal, and amplicon sequence variant (ASV) inference using DADA2 [11,12,13]. Taxonomic assignment was performed using a naïve Bayes classifier implemented in QIIME2 against the Greengenes reference database (Release 13.8) [14,15,16]. Alpha and beta diversity metrics were calculated based on ASV tables and visualized using principal coordinate analysis (PCoA) and non-metric multidimensional scaling (NMDS), with statistical significance assessed by PERMANOVA [17,18].

The sequencing depth ranged from 69,798 to 97,144 raw reads per sample, with an average of 85,964 reads per sample. To minimize biases caused by unequal sequencing depths among samples, the feature table was rarefied using the QIIME feature-table rarefy function, and the rarefaction depth was set to 95% of the sequence count of the sample with the lowest sequencing depth. Taxonomic assignment was performed using the Greengenes2 database (version 2022.10). The raw sequencing data have been deposited in the NCBI Sequence Read Archive (SRA) under accession number PRJNA1465754.

### 2.9. Data Analysis

All statistical analyses of intestinal morphology, serum biochemical parameters, immune indices, and antioxidant indices were performed using IBM SPSS Statistics 25.0 (IBM Corp., Armonk, NY, USA). Data are presented as mean ± standard deviation (SD). Differences among the four treatment groups were analyzed using one-way analysis of variance (ANOVA), followed by Duncan’s multiple range test for multiple comparisons. A value of *p* < 0.05 was considered statistically significant.

For gut microbiota analysis, alpha diversity indices were compared using Student’s *t*-test after confirming normality of the data. Beta diversity was evaluated based on the Bray–Curtis distance matrix and visualized by non-metric multidimensional scaling (NMDS) in R software (version 4.3.3). Differences in microbial community structure between groups were assessed using permutational multivariate analysis of variance (PERMANOVA). Differential taxa were identified using linear discriminant analysis effect size (LEfSe), with a linear discriminant analysis (LDA) score > 2.0 and *p* < 0.05 considered statistically significant.

For untargeted metabolomics, principal component analysis (PCA), partial least squares-discriminant analysis (PLS-DA), and differential metabolite analyses were performed using the Pasonno metabolomics analysis platform (Shanghai Pasonno Biotechnology Co., Ltd., Shanghai, China). Differential metabolites were identified based on the following criteria: a variable importance in projection (VIP) value > 1, a *p* value < 0.05, and a fold change (FC) > 1. Kyoto Encyclopedia of Genes and Genomes (KEGG) pathway enrichment analysis was subsequently performed to identify significantly enriched metabolic pathways.

## 3. Results

### 3.1. Effects of CEP on Intestinal Structure

**Figure 1:**
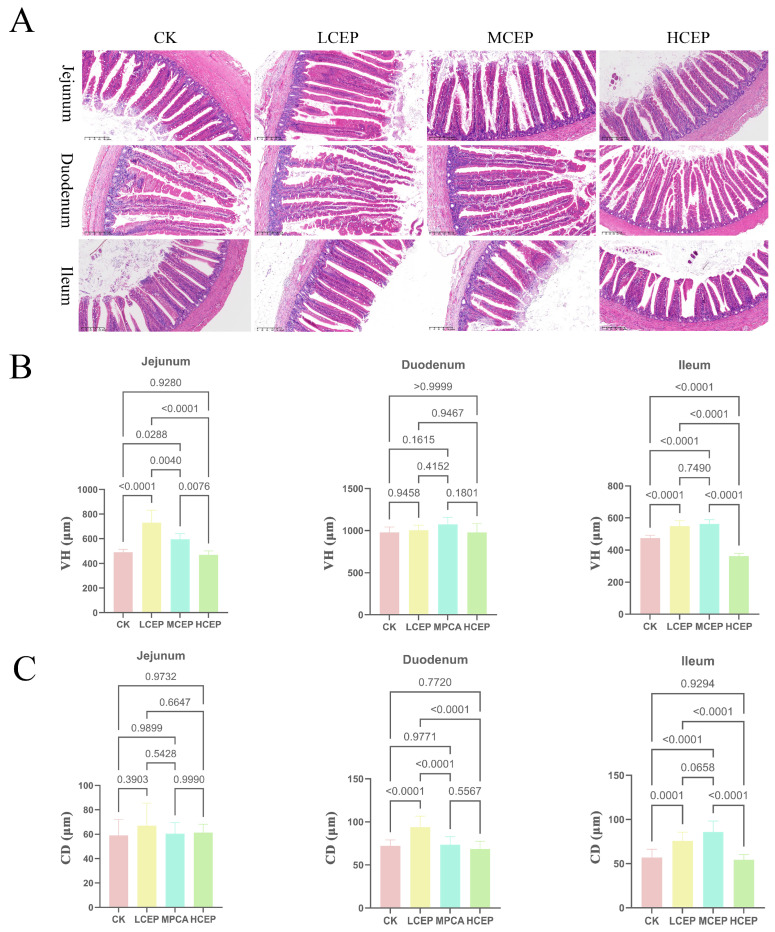
The effects of different dosages of the CEPs on intestinal morphology and digestive function in pigeons. (**A**) H&E staining of jejunal, duodenal and ileum tissue sections. (**B**) Villus height (VH) in the jejunum, duodenum and ileum. (**C**) Crypt depth (CD) in the jejunum, duodenum and ileum. CK group, control group; LCEP, low compound enzyme preparation group; MCEP, middle compound enzyme preparation group; HCEP, high compound enzyme preparation group.

**Figure 2:**
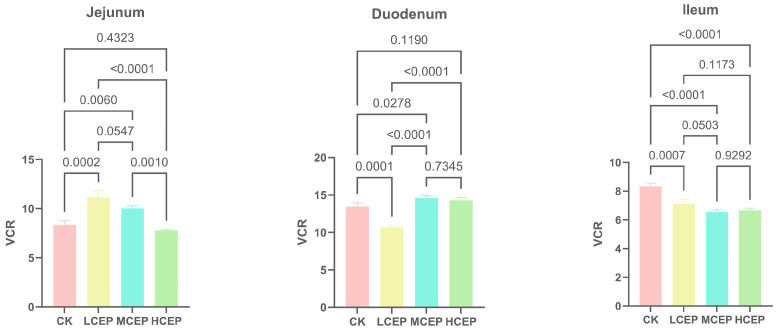
The effects of different dosages of the CEPs on intestinal morphology and digestive function in pigeons. VH to CD ratio (VCR) in the jejunum, duodenum and ileum. CK group, control group; LCEP, low compound enzyme preparation group; MCEP, middle compound enzyme preparation group; HCEP, high compound enzyme preparation group.

The results of H&E staining are shown in Figure 1A. This figure illustrates the impact of CEP supplementation on intestinal structural development, which is closely associated with nutrient digestion and absorption capacity in different segments of the small intestine. Results showed that LCEP group pigeons exhibited significantly higher VH in the jejunum compared to the other three groups (Figure 1B), while no significant differences in CD were observed between the control and experimental groups in the jejunum. In the duodenum, the LCEP group demonstrated significantly higher CD compared to the other three groups (Figure 1C), while no significant differences were observed between the control group and both experimental groups in VH (Figure 1B). Compared with the CK group, VCR in the jejunum was significantly reduced in all CEP-treated groups (*p* < 0.05). In contrast, the duodenal VCR was significantly increased in the LCEP and MCEP groups (*p* < 0.05), while no significant difference was observed between the CK and HCEP groups. In the ileum, the MCEP group exhibited the highest VCR, significantly exceeding those of the CK and LCEP groups (*p* < 0.05), whereas the HCEP group showed an intermediate level without significant differences from either the CK or MCEP group (Figure 2). The detailed statistical comparisons among all groups are summarized in Table A1.

### 3.2. Effects of CEPs on Biochemical Indicators and Immune Function

**Figure 3:**
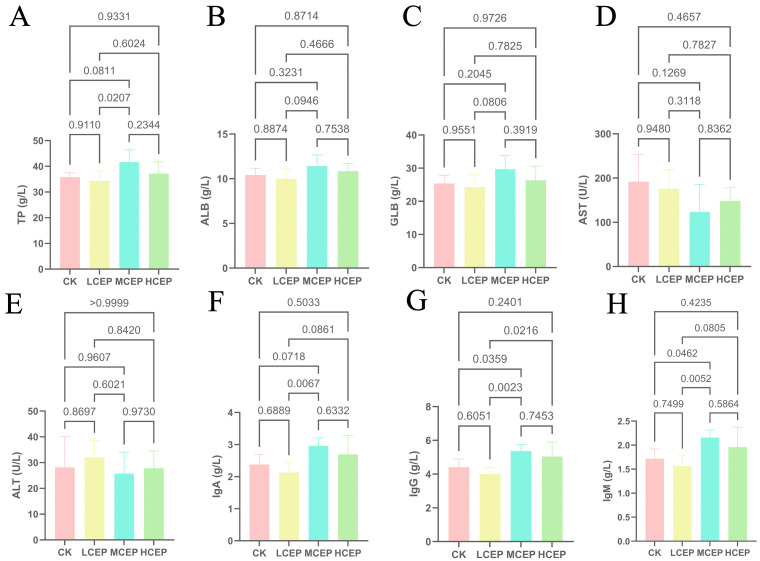
The effects of different dosages of the CEPs on the biochemical indicators of pigeon serum. (**A**) Total protein (TP, g/L); (**B**) albumin (ALB, g/L); (**C**) globulin (GLB, g/L); (**D**) aspartate aminotransferase (AST, U/L); (**E**) alanine aminotransferase (ALT, U/L); (**F**) immunoglobulin A (IgA, g/L); (**G**) immunoglobulin G (IgG, g/L); (**H**) immunoglobulin M (IgM, g/L). The data analysis was conducted using one-way ANOVA. The error bars represent the means ± standard deviation (SD) of six replicates. CK group, control group; LCEP, low compound enzyme preparation group; MCEP, middle compound enzyme preparation group; HCEP, high compound enzyme preparation group.

To investigate the effects of CEPs on serum biochemical parameters and immune status in pigeons, we collected blood samples from all pigeon groups and analyzed serum biochemical indicators and immune function. Results showed that no significant differences in ALB, GLB, AST, and ALT levels were observed in all groups (Figure 3B–E). In addition, the TP level was significantly higher in the MCEP group than in the LCEP group (Figure 3A). Additionally, compared with the control group, the IgA level was significantly decreased in the LCEP group, whereas the MCEP group showed significantly higher IgG and IgM levels. (Figure 3F–H). The detailed statistical comparisons among all groups are provided in Table A2.

### 3.3. Effects of CEPs on Antioxidant Capacity

**Figure 4:**
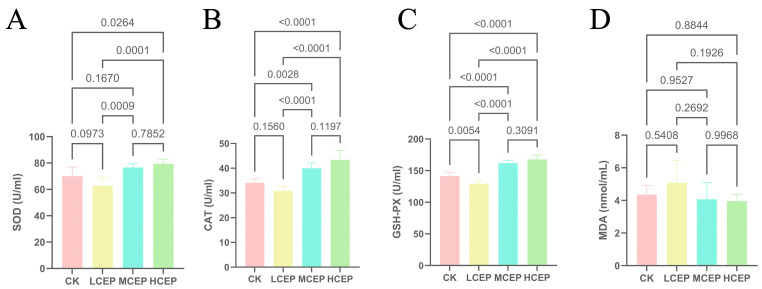
The effects of different dosages of the CEPs on the antioxidant indicators of pigeon serum. (**A**) Superoxide dismutase (SOD, U/mL); (**B**) catalase (CAT, U/mL); (**C**) glutathione peroxidase (GSH-Px, U/mL); (**D**) malondialdehyde (MDA, nmol/mL). The data analysis was conducted using one-way ANOVA. The error bars represent the means ± standard deviation (SD) of six replicates. CK group, control group; LCEP, low compound enzyme preparation group; MCEP, middle compound enzyme preparation group; HCEP, high compound enzyme preparation group.

To assess the impact of CEP on the antioxidant capacity, we detected the MDA levels and SOD, CAT, and GSH-Px activities. As shown in Figure 3, supplementing CEPs significantly increased the SOD activity in the HCEP group. The CAT activity was significantly increased in both the MCEP and HCEP groups. The GSH-Px level decreased significantly in the LCEP group, while it increased significantly in both the MCEP and HCEP groups. The MDA level showed no significant difference among all groups (Figure 4A–D). The detailed statistical comparisons among all groups are provided in Table A3.

### 3.4. Effects of CEPs on Intestinal Microbiota

**Figure 5:**
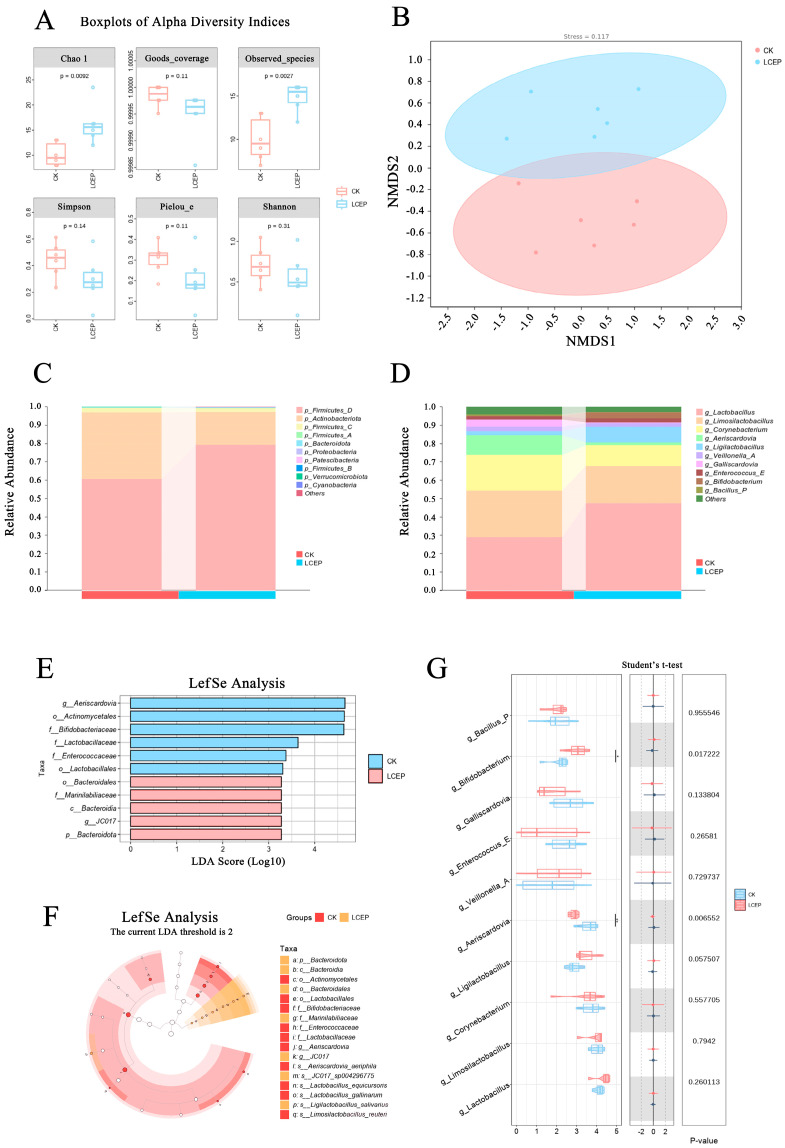
The effects of LCEP on the intestinal microbiota of pigeons. (**A**) Boxplots of alpha diversity indices of CK and LCEP groups. (**B**) Non-metric multidimensional scaling (NMDS) plot of CK and LCEP groups. (**C**) Taxa composition plot of CK and LCEP groups at the phylum level. (**D**) Taxa composition plot of CK and LCEP groups at the genus level. (**E**) Linear discriminant analysis (LDA) bar chart of CK and LCEP groups. (**F**) Linear discriminant analysis effect size (LEfSe) cladogram of CK and LCEP groups. (**G**) Species difference analysis plot of CK and LCEP groups. CK group, control group; LCEP, low compound enzyme preparation group.

After comprehensively analyzing the intestinal morphology, serum biochemical parameters, immunoglobulin, and antioxidant capacity, we found that the LCEP group yielded the most favorable results. Therefore, we conducted a microbial analysis of the ileum contents samples from both the CK and LCEP groups to investigate the impact of CEPs on the microbial structure in the ileum. The alpha diversity analyses show that both the Chao1 and the Observed species indicators were upregulated (*p* < 0.05) in the LCEP group compared with the CK group (Figure 5A). The NMDS analysis revealed a clear difference in the composition of the intestinal microbiota between the CK group and the LCEP group (Figure 5B). At the phylum level, *p_Bacteroidota* was significantly upregulated, while at the genus level, *g_JC017* (an unclassified genus within the family Marinilabiliaceae) was significantly upregulated (Figure 5C,D). The LEfSe method was used to analyze the taxa with rich differences between the CK group and the LCEP group (*p* < 0.05, LDA score > 2). Taxa enriched in CK group mainly include *g_Aeriscardovia*, *o_Actinomycetales*, *f_Bifidobacteriaceae*, *f_Lactobacillaceae*, *f_Enterococcaceae*, and *o_Lactobacillales* (*p* < 0.05), while in LCEP group are *o_Bacteroidale*, *f_Marinilabiliaceae*, *c_Bacteroidia*, *g_JC017*, and *p_Bacteroidota* (*p* < 0.05) (Figure 5E,F). Meanwhile, at the genus level, the number of *g_Bifidobacterium* was significantly increased in the LCEP group compared with the CK group (*p* < 0.05) (Figure 5G).

### 3.5. Effects of CEP on Intestinal Metabolites

**Figure 6:**
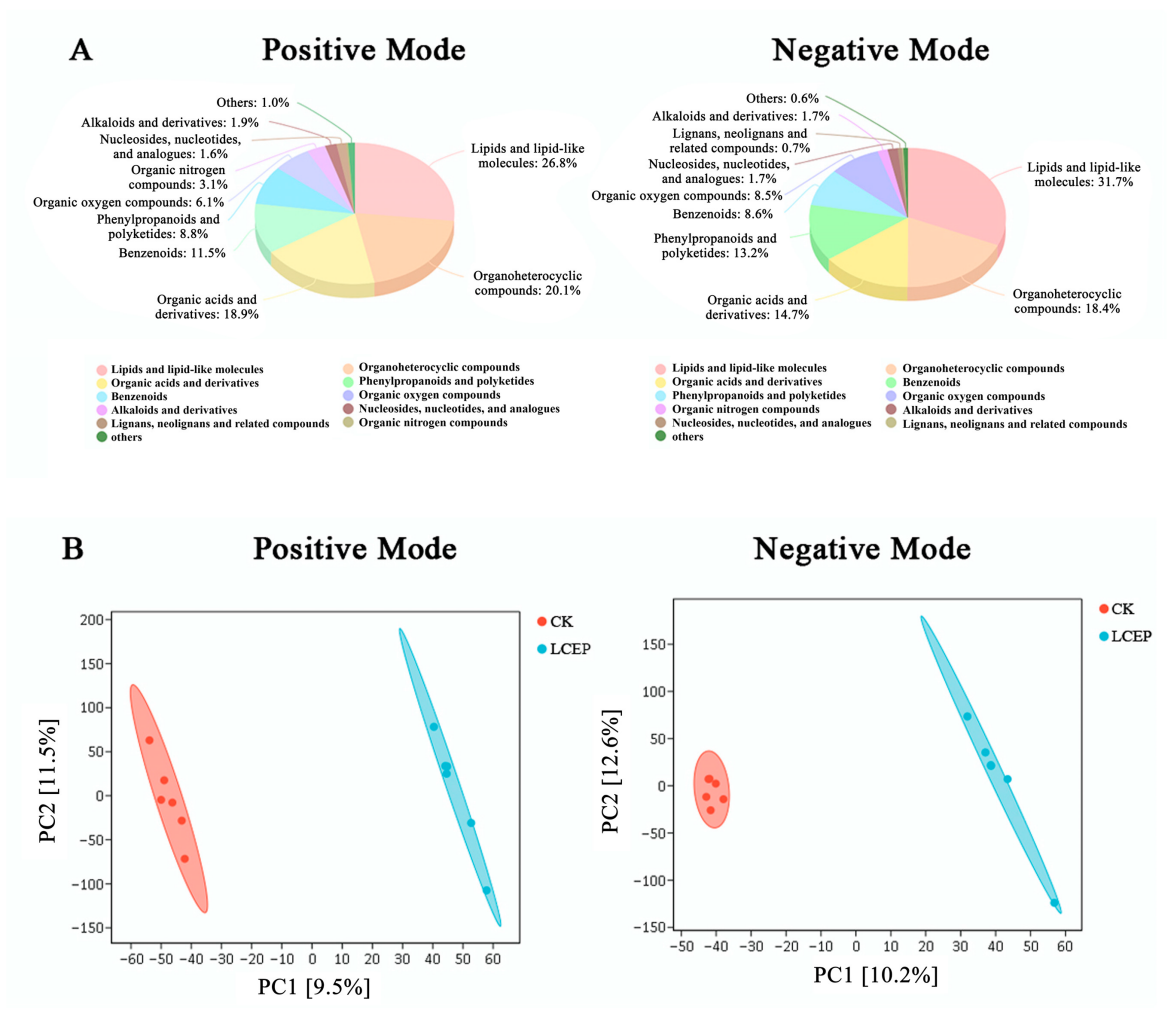

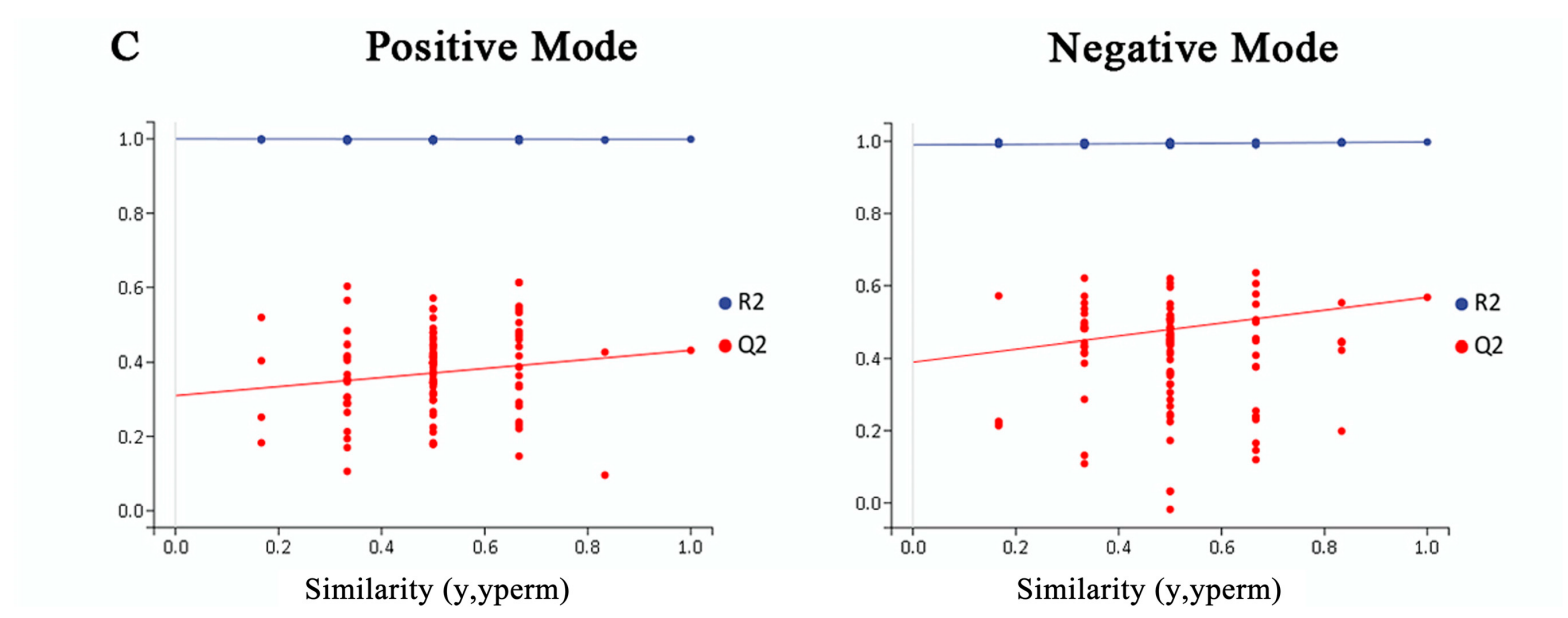
The effects of CEPs on the intestinal metabolites of pigeons. (**A**) Categorized pie charts for metabolite identification analysis of CK and LCEP groups in positive mode and negative mode. (**B**) Partial least squares-discriminant analysis (PLS-DA) score plot of the CK and LCEP groups in positive mode and negative mode. (**C**) Model interpretability (R2Y) and predictability (Q2) for positive and negative modes. CK group, control group; LCEP, low compound enzyme preparation group.

**Figure 7:**
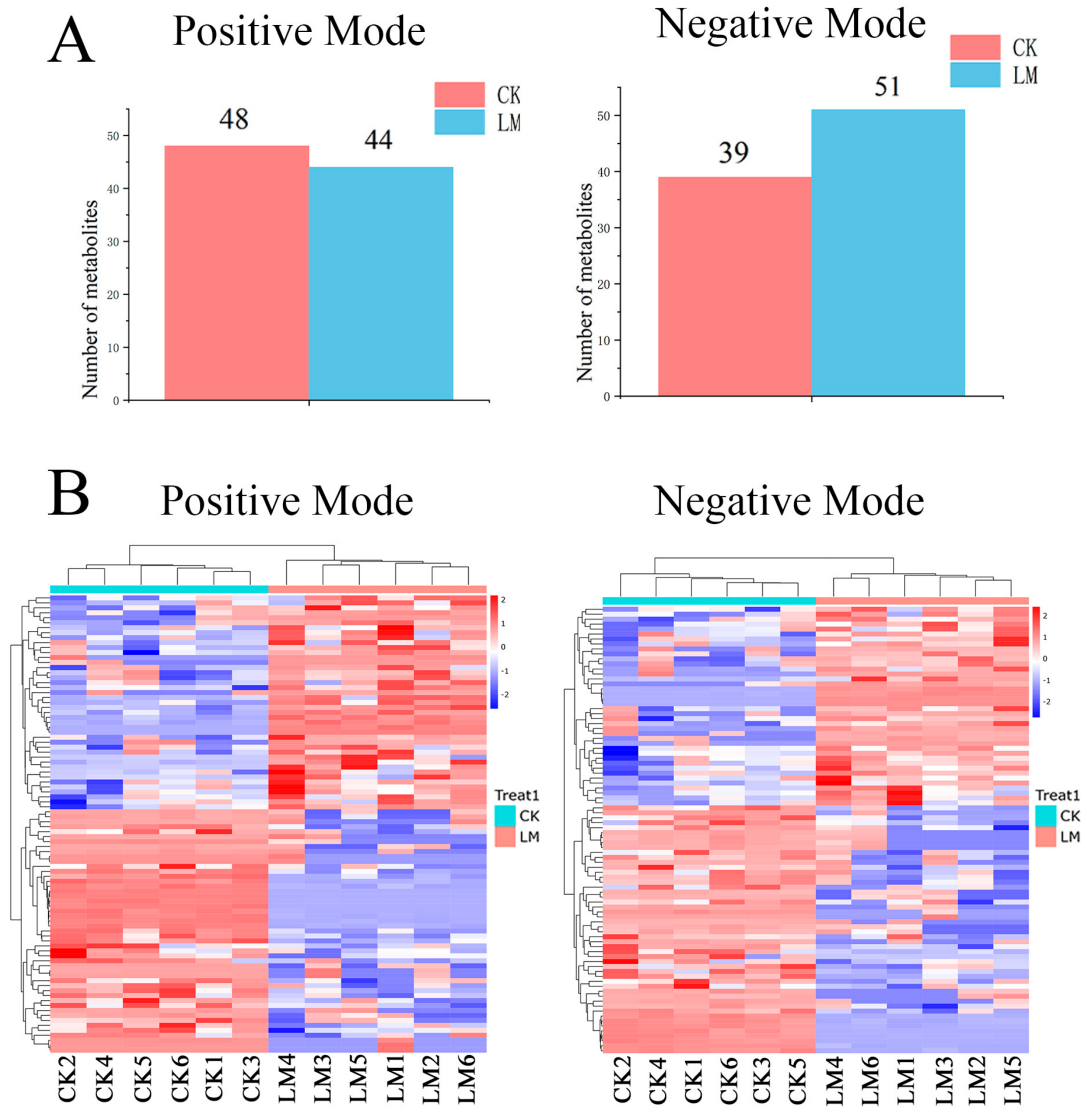

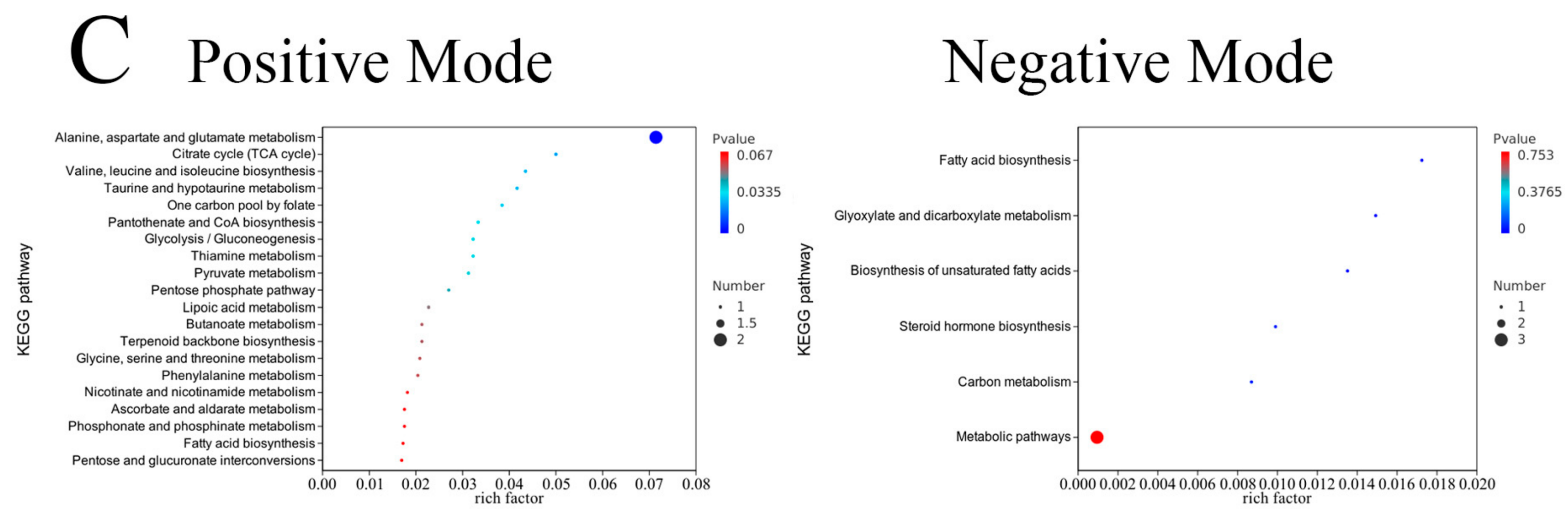
The effects of CEPs on the intestinal metabolites of pigeons. (**A**) Differential substance screening chart of CK and LCEP groups in positive mode and negative mode. (**B**) Clustering heat map of differential substance enrichment analysis of CK and LCEP groups in positive mode and negative mode. (**C**) Kyoto Encyclopedia of Genes and Genomes (KEGG) enrichment pathways of CK and LCEP groups in positive and negative mode. CK group, control group; LCEP, low compound enzyme preparation group.

We further measured the levels of metabolites in the ileum contents of the CK and LCEP groups. The results are shown in Figure 6. In the positive mode, the five components with the highest contents were lipids and lipid-like molecules (26.8%), organoheterocyclic compounds (20.1%), organic acids and derivatives (18.9%), benzenoids (11.5%), and phenylpropanoids and polyketides (8.8%), while in the negative mode, were lipids and lipid-like molecules (31.7%), organoheterocyclic compounds (18.4%), organic acids and derivatives (14.7%), phenylpropanoids and polyketides (13.2%), and benzenoids (8.6%) showing the highest (Figure 6A). Then, partial least squares-discriminant analysis (PLS-DA) models in both positive and negative modes were analyzed. The PLS-DA results indicated significant differences between the CK and the CEP groups (Figure 6B). Subsequent permutation tests confirmed that the model had good fitting and predictive capabilities, and no outliers were observed (Figure 6C). Forty-eight metabolites were upregulated and 44 were downregulated in the positive mode compared with the control group, while 39 metabolites were upregulated and 51 were downregulated in the negative mode (Figure 7A). Moreover, significant differences in metabolites were observed between the CK and the LCEP group by the clustering heat map of differential substance enrichment analysis in both positive and negative modes (Figure 7B). KEGG analysis indicated that in LCEP group, differential metabolites were enriched in alanine, aspartate and glutamate metabolism, citrate cycle (TCA cycle), valine, leucine and isoleucine biosynthesis, taurine and hypotaurine metabolism, and one-carbon pool by folate in positive mode, while fatty acid biosynthesis, glyoxylate and dicarboxylate metabolism, biosynthesis of unsaturated fatty acids, steroid hormone biosynthesis, and carbon metabolism were enriched in negative mode (Figure 7C).

## 4. Discussion

The digestive system of pigeons is unable to produce cellulase and thus cannot degrade cellulose, hemicellulose, and other NSPs in plant cell walls. These indigestible structures physically entrap proteins, starch, lipids, and other nutrients, thereby reducing their digestibility. As a result, the inability to utilize NSPs and other anti-nutritional factors reduces feed utilization and ultimately affects production performance and economic efficiency [19].

The CEP has broad application prospects in livestock and poultry feed. It has a relatively high enzymatic activity, and can synergistically decompose non-starch polysaccharides, phytic acid, macromolecular proteins, starch and other components in feed, thereby improving the release efficiency and availability of nutrients in feed [20]. A previous study found that adding CEP to basal diet for laying hens significantly increased egg production and reduced feed-to-egg ratio; meanwhile, improved feed digestibility and nutrient conversion efficiency has promoted the enhancement of production performance [21].

The small intestine is a major digestive site in the animals, and the morphology of the small intestine can serve as an indicator of intestinal health and integrity [22]. Small intestinal villi can greatly increase the inner surface area of the small intestine, thereby efficiently absorbing nutrients after food digestion. Longer small intestinal villi have a larger surface area and thus have a stronger absorption capacity. Conversely, shorter small intestinal villi have a relatively weaker digestive capacity compared to long villi [23]. Crypts can be regarded as the “factories” of villi, and an increased crypt depth may reflect enhanced epithelial cell proliferation and tissue renewal [24]. However, under certain conditions, it may also be associated with intestinal inflammation. Therefore, crypt depth should be interpreted together with other intestinal morphological parameters, particularly the villus height-to-crypt depth ratio. The balance between these two parameters is essential for maintaining intestinal health, and the VCR is directly dependent on the relationship between VH and CD [25].

An increased VCR generally reflects improved absorptive function, whereas a decreased ratio suggests mucosal damage or increased epithelial repair pressure [22]. Interestingly, supplementation with 0.25 g/kg of CEPs increased the VCR in the jejunum but decreased it in the duodenum, indicating that the effects of CEPs on different segments of the small intestine are not consistent. This differential effect may be attributed to the distinct physiological roles of the duodenum and jejunum. The duodenum, being the primary site for chyme neutralization and initial enzymatic digestion, is exposed to higher concentrations of endogenous digestive secretions and experiences a more dynamic luminal environment compared with the jejunum. Consequently, the lower dose (0.25 g/kg) of CEPs may be insufficient to adequately optimize the digestive microenvironment in the duodenum, whereas this dose already appears adequate to enhance nutrient digestion and absorption in the jejunum, thereby promoting improved mucosal morphology in this segment. When the supplementation dose was increased to 0.50 g/kg, the exogenous enzyme content was further elevated, potentially reaching a level sufficient to exert beneficial effects throughout the proximal small intestine, thus ameliorating duodenal histomorphology. Nevertheless, given that parameters such as digestive enzyme activities, nutrient digestibility, and intestinal epithelial cell proliferation were not measured in the present study, the above explanations remain speculative and warrant further investigation for validation.

Metabolomic analysis showed a significant increase in the folate levels among intestinal metabolites, and upregulation of the one-carbon pool by folate pathway was observed in the LCEP group. This pathway provides essential one-carbon units for the synthesis of purines and thymidylate—the building blocks of DNA—thereby influencing nucleotide metabolism and DNA integrity in the intestine [26]. These processes are particularly important for rapidly dividing cells [27], such as intestinal epithelial cells. Therefore, we speculate that the activation of folate-mediated one-carbon metabolism may have contributed to the improved intestinal morphology observed in the LCEP group. However, this potential mechanism is inferred from the metabolomic data and requires further experimental validation.

The result of serum biochemical indicators revealed a significant increase in TP in the MCEP group compared with the LCEP group, while no significant changes were observed in ALB, GLB, AST, and ALT. TP is the sum of all proteins in the serum, mainly albumin and globulins. This result shows the improvement of the state of digestion and absorption of proteins without liver stress [28]. Another study also shows that adding multi-enzyme preparations to drinking water can increase TP in broiler plasma with a change in ALB, globulin, or the activity of liver enzymes, which also indicates that protein assimilation is enhanced rather than liver cell damage [29]. Furthermore, we also determined the immune and antioxidant indicators in the serum of each group. The immunological results showed that the CK group exhibited significant differences in the three indicators of IgA, IgM, and IgG. The IgM is the first antibody isotype produced in response to initial antigen exposure, indicating the early or primary immune initiation [30]. During a secondary immune response, IgG, derived from IgM and IgD through class-switch recombination, predominates and provides long-term protection [31]. IgA is the main immunoglobulin on the mucosal surface, which plays a critical role in interacting with the microbiota and maintaining intestinal homeostasis [32]. The increasing level of IgA in serum may indicate that the CEP enhanced mucosal immune activity or the transfer of IgA from mucosal tissue to the bloodstream. The observed changes in serum immunoglobulin levels suggest that CEP supplementation may modulate the humoral immune response in pigeons.

Antioxidant capacity is of vital importance as it reflects the ability to neutralize reactive oxygen species (ROS) produced during normal metabolism and stress processes, preventing DNA, proteins, and lipids from oxidative damage. Maintaining a formidable antioxidant capacity can protect cells from oxidative stress-induced damage and help maintain overall physical health [33]. SOD is an enzymatic antioxidant that protects cells from damage caused by superoxide radicals. CAT is an antioxidant enzyme that breaks down hydrogen peroxide into water and oxygen, protecting cells from peroxide-induced oxidative injury. GSH-Px is an antioxidant enzyme that uses reduced glutathione (GSH) as a substrate. It reduces hydrogen peroxide and lipid peroxides to non-toxic products. MDA is a stable end-product of lipid peroxidation. It reflects the degree of oxidative damage to polyunsaturated fatty acids in cell membranes. Our results indicated an increase in SOD, CAT, and GSH-Px, while there was a declining trend in MDA in the MCEP and HCEP groups. These results demonstrate that CEP supplementation enhanced antioxidant defense capacity, reflecting improved redox homeostasis and alleviating oxidative stress.

Intestinal microbiota is a key component of the digestive system. To understand if the CEP affects the intestinal microbiota, we sequenced the samples and found that the intestinal microbiota has improved, and the species richness has significantly increased in the LCEP group compared with the CK group. At the phylum level, *p_Bacteroidota* has significantly increased, and *g_Bifidobacterium* also increased at the genus level. *p_Bacteroidota* plays an important role in the degradation of compound polysaccharides and the production of short-chain fatty acids (SCFAs) [34]. In the present study, the increased abundance of *p_Bacteroidota* and *g_Bifidobacterium* suggests an enhanced capacity for carbohydrate fermentation and SCFA production. Previous studies have shown that SCFAs contribute to intestinal homeostasis by inhibiting pathogenic bacteria, providing energy for intestinal epithelial cells, and maintaining intestinal barrier integrity [35,36,37]. Therefore, the enrichment of these taxa may have contributed to the improved intestinal health observed in the CEP-treated pigeons.

Although SCFAs were not directly quantified in the present study, the increased relative abundance of bifidobacterium and bacteroidota may suggest an enhanced capacity for carbohydrate fermentation, as these taxa are widely recognized as important contributors to SCFA production. Therefore, the beneficial effects of CEPs on intestinal health observed in this study may be mediated, at least in part, through microbiota-associated metabolic functions. However, this hypothesis requires further validation by direct quantification of SCFAs in future studies.

Interestingly, although CEP supplements usually improve intestinal health, higher supplement doses do not further enhance their beneficial effects. This observation shows that the effect of CEPs may be dose-dependent. One possible explanation is that excessive enzyme supplementation may cause the degradation of non-starchy polysaccharides to exceed the optimal level, thus reducing the fermentation substrate available to beneficial intestinal microorganisms. In addition, the excessive hydrolysis of NSPs may change the physicochemical characteristics of intestinal digesters, such as viscosity and fermentation mode, thereby weakening the favorable interaction between the intestinal flora and the host. However, these potential mechanisms are still speculative and require further research.

## 5. Limitations

Despite these promising findings, several limitations of the present study should be acknowledged. First, the primary objective of this study was to investigate the effects of CEPs on intestinal health and the associated changes in the gut microbiota and metabolome. Therefore, growth performance parameters, such as average daily feed intake, average daily gain, and feed conversion ratio, were not included in the experimental design. Future studies incorporating these production performance indicators will provide a more comprehensive evaluation of the practical benefits of CEP supplementation. Second, due to the substantial cost and resource requirements associated with multi-omics analyses, only the CK and LCEP groups were included in the microbiome and metabolomic analyses. Therefore, these findings should be interpreted as comparisons between the CK and LCEP groups, and further studies including all treatment groups are warranted to validate the dose-dependent effects of CEP supplementation. Third, although changes in the gut microbiota suggested a potential enhancement of microbial metabolic functions, short-chain fatty acids were not directly quantified. Therefore, the proposed role of SCFAs in mediating the beneficial effects of CEPs remains speculative and requires further validation. Finally, while untargeted metabolomics identified multiple differential metabolites associated with CEP supplementation, the causal relationships between specific microbial taxa, metabolites, and improvements in intestinal health require further investigation through targeted metabolomics and functional studies.

## 6. Conclusions

In summary, this study showed that dietary CEP supplementation could improve antioxidant capacity and immune response. Based on the overall findings of this study, 0.25 g/kg of CEPs showed the most consistent beneficial effects on intestinal health and was associated with favorable changes in the gut microbiota and metabolome. However, different supplementation levels exerted the greatest effects on different physiological parameters, and further studies are needed to determine the optimal dosage under different production objectives. Collectively, we provided a theoretical basis for the development of CEPs as a feed additive for pigeons.

## Figures and Tables

**Table 1:** Composition and nutrient levels of the basal diet (air-dry basis).

Ingredient	Content (%)	Nutrient Component ^2^	Content (%)
Corn	53.00	ME (MJ/kg)	12.81
Pea	30.00	CP (%)	12.90
Wheat	7.00	Lysine (%)	1.05
Sorghum	7.00	Methionine (%)	0.44
NaCl	0.50	Ca (%)	1.20
Limestone	1.50	Available Phosphorus (AP) (%)	0.54
Premix ^1^	1.00		
Total	100.00		

^1^ The premix provided the following per kg diet: vitamin A, 6000.00 IU; vitamin D, 1200.00 IU; vitamin E, 34.00 1U; vitamin B_1_, 3.2 mg; vitamin B_2_, 7.20 mg; vitamin B_12_, 0.10 mg; biotin, 1.34 mg; folic acid, 0.55 mg; nicotinic acid, 15.00 mg: pantothenic acid, 7.50 mg; Cu (CuSO_4_), 12.00 mg; Fe (FeSO_4_), 35.00 mg; Zn (ZnSO_4_), 35.00 mg; Mn (MnSO_4_), 55. 00 mg; I (KI), 0.25 mg. ^2^ ME was a calculated value, while the other were measured values.

## Data Availability

The original data presented in the study are openly available in NCBI at PRJNA1465754.

## Supplementary Materials

The following supporting information can be downloaded at: https://www.mdpi.com/article/10.3390/ani16152353/s1.

## Appendix A

**Table A1:** Villus height (VH), crypt depth (CD), and villus height-to-crypt depth ratio (VCR) of the ileum, duodenum, and jejunum.

Indicators	Jejunum				Duodenum				Ileum			
	CK	LCEP	MCEP	HCEP	CK	LCEP	MCEP	HCEP	CK	LCEP	MCEP	HCEP
VH (μm)	491.00 ± 24.71 ^c^	730.70 ± 101.50 ^a^	595.60 ± 45.28 ^b^	470.30 ± 31.86 ^c^	978.40 ± 64.16	1003.00 ± 62.12	1075.00 ± 83.20	977.60 ± 104.10	473.60 ± 18.18 ^b^	548.40 ± 34.65 ^a^	562.20 ± 25.95 ^a^	362.20 ± 18.15 ^c^
CD (μm)	58.97 ± 13.2	67.02 ± 18.56	60.54 ± 9.01	61.25 ± 6.92	72.09 ± 6.99 ^b^	93.84 ± 12.68 ^a^	73.62 ± 9.14 ^b^	68.48 ± 8.95 ^b^	56.77 ± 9.56 ^b^	75.64 ± 10.01 ^a^	85.82 ± 12.40 ^a^	54.35 ± 6.10 ^b^
VCR	8.33 ± 0.45 ^b^	11.14 ± 0.68 ^a^	10.02 ± 0.27 ^b^	7.765 ± 0.12 ^b^	13.44 ± 0.53 ^b^	10.69 ± 0.28 ^c^	14.61 ± 0.36 ^a^	14.27 ± 0.38 ^ab^	8.33 ± 0.22 ^a^	7.13 ± 0.32 ^b^	6.55 ± 0.15 ^b^	6.66 ± 0.15 ^b^

Note: One-way analysis of variance (ANOVA) was performed. Data in the table are presented as mean ± standard deviation (SD). Differences were considered statistically significant at *p* < 0.05, and different superscript letters indicate significant differences among groups.

**Table A2:** Effects of the CEPs on the biochemical and immune indicators.

Indicators	CK	LCEP	MCEP	HCEP
TP (g/L)	35.79 ± 1.83 ^ab^	34.27 ± 3.90 ^b^	41.65 ± 4.80 ^a^	37.15 ± 4.64 ^ab^
ALB (g/L)	10.40 ± 0.76	9.98 ± 1.10	11.42 ± 1.25	10.84 ± 0.85
GLB (g/L)	25.38 ± 2.44	24.29 ± 3.70	29.75 ± 4.04	26.30 ± 4.31
AST (U/L)	191.80 ± 61.60	175.90 ± 42.77	123.60 ± 62.02	148.20 ± 30.30
ALT (U/L)	28.16 ± 11.92	31.99 ± 6.61	25.71 ± 8.39	27.86 ± 6.71
IgA (g/L)	2.38 ± 0.31 ^ab^	2.13 ± 0.30 ^b^	2.96 ± 0.25 ^a^	2.69 ± 0.59^ab^
IgG (g/L)	4.41 ± 0.48 ^bc^	4.01 ± 0.39 ^c^	5.37 ± 0.38 ^a^	5.05 ± 0.86 ^ab^
IgM (g/L)	1.72 ± 0.20 ^b^	1.56 ± 0.22 ^b^	2.16 ± 0.16 ^a^	1.96 ± 0.42 ^ab^

Note: One-way analysis of variance (ANOVA) was performed. Data in the table are presented as mean ± standard deviation (SD). Differences were considered statistically significant at *p* < 0.05, and different superscript letters indicate significant differences among groups.

**Table A3:** Effects of CEPs on the antioxidant capacity.

Indicators	CK	LCEP	MCEP	HCEP
SOD (U/mL)	70.04 ± 6.85 ^bc^	62.59 ± 6.59 ^c^	76.60 ± 2.83 ^ab^	79.44 ± 3.41 ^a^
CAT (U/mL)	34.06 ± 1.63 ^b^	30.87 ± 1.74 ^b^	40.01 ± 2.19 ^a^	43.41 ± 3.83 ^a^
GSH-Px (U/mL)	141.40 ± 5.54 ^b^	129.00 ± 5.05 ^c^	161.90 ± 4.69 ^a^	167.70 ± 6.95 ^a^
MDA (mmol/mL)	4.35 ± 0.58	5.08 ± 1.37	4.07 ± 1.04	3.96 ± 0.41

Note: One-way analysis of variance (ANOVA) was performed. Data in the table are presented as mean ± standard deviation (SD). Differences were considered statistically significant at *p* < 0.05, and different superscript letters indicate significant differences among groups.
