# Supplementary material for: Compound Enzyme Supplementation Improves Intestinal Health, Immunity, and Antioxidant Capacity in Pigeons via Gut Microbiota and Metabolome Modulation
Source: Animals (Basel). 2026 Aug 2;16(15):2353. doi: 10.3390/ani16152353 (PMC13463598; doi:10.3390/ani16152353)
Supplement: Supplementary file 1 [file animals-16-02353-s001.zip › animals-4378186-supplementary.pdf]

Poultry-Specific Compound Enzyme (Jiyoubao)

Product Description

Poultry have a relatively short digestive tract and rapid digesta passage, with feed typically excreted within 5–6 hours. The short retention time limits digestion and nutrient absorption, leading to undigested feed in feces or abnormal microbial fermentation in the intestine, which may result in watery droppings. Poor digestion can cause digestive disorders, increase disease incidence, and waste feed.

Product Features: Specifically formulated according to the digestive physiology of broilers and the nutritional characteristics of poultry feed. It enhances digestive capacity in broilers, reduces digestive tract disorders, improves undigested feed and watery droppings, improves flock health and uniformity, reduces morbidity and mortality, lowers feed conversion ratio, increases market weight, and improves production efficiency.

1. Produced using an advanced deep-fermentation process to obtain highly purified enzymes. A scientifically optimized multi-enzyme complex synergistically degrades macromolecules into easily digestible nutrients. Enzymes targeting anti-nutritional factors in plant ingredients disrupt the viscous polysaccharide network of plant cell walls, increasing nutrient contact with the intestinal mucosa, enhancing absorption, and reducing undigested feed and abnormal intestinal fermentation that cause watery droppings.

2. Contains microbial fermentation metabolites to enhance substrate nutrition, improve synergistic enzyme efficacy, supplement animal nutrition, promote digestion in broilers, enhance disease resistance, and reduce mortality and culling.

Product Composition

Cellulase, xylanase, compound protease, mannanase, amylase, pectinase, etc.

Directions for Use

Recommended inclusion: 500 g per metric ton of complete feed. The inclusion level may be increased according to flock health and digestive status.

Compatibility: May be used together with glucose oxidase, lysozyme, or similar products for routine intestinal health management, maintaining gut microbial balance, and alleviating bacterial diarrhea.

Applications

1. Improves feed digestibility and nutrient utilization, reduces undigested feed in feces, and lowers feed costs.
2. Reduces digesta viscosity, watery droppings, and vent pasting.
3. Regulates intestinal microbiota, reduces digestive tract diseases, and lowers mortality and culling rates.
4. Improves intestinal health, enhances disease resistance, and increases flock uniformity.

Packaging and Storage

Package: 500 g/bag

Shelf life: 12 months in the original sealed package when stored in a cool and dry environment.

Precautions

Avoid contact with mucous membranes or skin during handling. If contact occurs, rinse immediately with clean water.

As enzyme preparations are biologically active products, avoid exposure to rain and direct sunlight. Store in a cool, dry, and well-ventilated place. Use promptly after opening; if not used completely, reseal the bag tightly.
